# Association of Activating *GNAS* Mutations and Outcomes with Chemotherapy in Metastatic Appendiceal Adenocarcinoma

**DOI:** 10.1245/s10434-025-18805-5

**Published:** 2026-02-03

**Authors:** Rushabh Gujarathi, Christopher Rodman, Varun Vivek Bansal, Erika Belmont, Namrata Setia, Lindsay Alpert, John Hart, Mecker G. Möller, Oliver S. Eng, Grace Lee, Blase N. Polite, Kiran K. Turaga, Ardaman Shergill

**Affiliations:** 1 Department of Medicine, University of Louisville, Louisville, KY USA; 2 Department of Medicine, Yale School of Medicine, New Haven, CT USA; 3 Department of Surgery, University of Colorado, Aurora, CO USA; 4 Department of Medicine, Washington University School of Medicine, St Louis, MO USA; 5 Department of Pathology, University of Chicago, Chicago, IL USA; 6 Department of Surgery, University of Chicago, Chicago, IL USA; 7 Department of Surgery, University of California, Irvine, Orange, CA USA; 8 Department of Radiology, University of Chicago, Chicago, IL USA; 9 Department of Medicine, Section of Hematology/Oncology, University of Chicago, University of Chicago Medicine, Chicago, IL USA; 10 Department of Surgery, Yale School of Medicine, New Haven, CT USA

## Abstract

**Background:**

Findings have linked *GNAS*-activating mutations, frequent in appendiceal adenocarcinoma (AA), with improved overall survival but poor response to chemotherapy. The authors hypothesized that *GNAS*-activating mutations are associated with differential outcomes in AA treated with chemotherapy.

**Methods:**

Patients seen at the authors’ center between 2013 and 2023 who received systemic chemotherapy for metastatic/recurrent AA were identified. The primary outcome was disease event-free survival (EFS), defined as time from start of chemotherapy (5-fluorouracil/capecitabine based) to earliest disease event, including death, clinical/radiographic recurrence, or progression. Study outcomes were assessed using Kaplan-Meier estimations and Cox proportional hazards regression.

**Results:**

The study included 48 patients. In 18 (37.5 %) of the 48 patients, *GNAS*-activating mutations were seen. Patients with *GNAS* mutations were more likely to have lower grades of disease (*p* = 0.003), with lower proportions of lymphovascular invasion (*p* = 0.005) and perineural invasion (*p* = 0.03), but a higher median peritoneal carcinomatosis index (*p* = 0.03). In the multivariable analysis, *GNAS* mutations (10.7 months [95 % confidence interval {CI}, 7.1–19.2] vs 20.3 months [95 % CI, 18.6–29.4; adjusted HR {aHR}, 3.75; 95 % CI, 1.84–7.63] *p* < 0.001) and metachronous metastases (aHR, 5.14; 95 % CI, 2.08–12.69; *p* < 0.001) were associated with worse EFS. Both CC0-1 resection (aHR, 0.12; 95 % CI, 0.05–0.28; *p* < 0.001) and CC2-3 resection (aHR, 0.28; 95 % CI, 0.10–0.81; *p* = 0.02) were associated with prolonged EFS. There was no significant difference in the OS from the date of metastases diagnosis between the *GNAS*^mt^ and *GNAS*^wt^ patients (HR, 0.68; 95 % CI, 0.31–1.47; *p* = 0.33).

**Conclusions:**

With systemic chemotherapy, *GNAS*-mutated metastatic/recurrent AAs have worse EFS despite less frequent high-risk features. Routine somatic mutation-testing of patients with AA should be considered for prognostication and possibly therapeutic decision-making.

**Supplementary Information:**

The online version contains supplementary material available at 10.1245/s10434-025-18805-5.

Appendiceal cancers (ACs), rare tumors with an incidence of approximately 1.72 cases per 100,000 person-years, exhibit significant histologic diversity.^1–3^ The various histologic subtypes include well-differentiated neuroendocrine tumors, mucinous neoplasms, goblet cell adenocarcinomas, colonic-type adenocarcinomas, and signet ring cell adenocarcinomas.^2^ Appendiceal adenocarcinoma (AA) variants collectively comprise 40 % to 64 % of all appendiceal cancers.^2–4^ Conventionally, the histologic subtype of the tumor is considered the key determinant in guiding clinical management and informing prognosis.^2,5–7^

However, the current histologic classification may have several shortcomings in accurately defining the prognosis and predicting clinical outcomes for patients with AA.^8^ Although tumor grade is considered to be predictive of survival, a great degree of variability is often noted in disease aggressiveness, chemotherapy responsiveness, and ultimately overall survival (OS) within patients with similar histopathologic features.^8–12^

Tumor molecular-profiling, including *RAS* and *BRAF* alterations in colorectal cancer, has proved to be instrumental in identifying new treatment avenues and in predicting and prognosticating therapeutic susceptibilities.^13^ Molecular profiling of AAs has shown these tumors to exhibit distinct molecular traits compared with colorectal cancers, together with molecular diversity across their histopathologic subtypes.^14^

The *GNAS* (GNAS complex locus) is a proto-oncogene coding for the Gsα component of complex G-proteins, which play a role in cellular cyclic AMP (cAMP) production.^15^ In various epithelial neoplasms, *GNAS* hotspot mutations are seen, particularly in codons 201 and 227,^15–17^ leading to constitutive activation of the G-stimulatory pathway and resulting in an increase in cellular cAMP production.^16^ Solid tumors with *GNAS* mutations display a consistent molecular and clinical phenotype characterized by mucinous tumor characteristics, an increased incidence of peritoneal metastasis, reduced efficacy of first-line systemic therapies, and poorer survival outcomes.^18^ *GNAS* mutations are particularly common in appendiceal neoplasms and seen in up to 52 % of AAs, depending on the histologic subtype.^14,19,20^

In a study involving 15 patients with low- or high-grade pseudomyxoma peritonei (PMP) of appendiceal origin who were treated with metronomic capecitabine for disease relapse and progressive disease after cytoreductive surgery (CRS), *GNAS* mutations were associated with shorter progression-free survival (PFS).^21^ These results were further validated by the study authors using outcomes from a previous cohort of 11 patients with PMP of appendiceal origin treated with 5-fluorouracil-based chemotherapy (FOLFOX-4), in which *GNAS* mutations were associated with shorter PFS.^21^

Furthermore, the analysis of Foote et al.^8^ has identified a potentially “chemotherapy-resistant *GNAS*-mut predominant subtype” of AAs, further highlighting the importance of conducting somatic tumor profiling for this rare malignancy. Therefore, we hypothesized that these mutations may have a role to play in determining treatment outcomes for patients with metastatic/recurrent AA who receive 5-fluorouracil/capecitabine-based chemotherapy with or without CRS, both of which comprise the cornerstones of the clinical management of AA.^22^ Our retrospective analysis aimed to explore the impact of these mutations on disease outcomes after reception of chemotherapy.

## Materials and Methods

### Study Subjects

This study comprised a retrospective review of electronic medical records of consecutive patients with AA seen at the University of Chicago between November 2013 and November 2023. The review of records was conducted under the purview of an institutional review board protocol approved at the University of Chicago (IRB24-0934). Given the retrospective nature of review of records, a waiver of consent was granted. Patients with histopathologic confirmation of metastatic AA and tumor next-generation sequencing (NGS) results available were included in the study cohort. Patients with bothsynchronous and metachronous metastases were included.

### *Inclusion and Exclusion Criteriae*

The study included patients with AA including the mucinous, goblet cell, signet ring cell, and colorectal subtypes. Patients should have received a minimum of 3 months of systemic chemotherapy for metastatic disease with 5-fluorouracil/capecitabine-based regimens for inclusion in this study.. Patients who had received chemotherapy for locoregional disease in the preceding 6 months were excluded. This criteria served to exclude cases in which metastatic disease might have been present at the initial diagnosis but had a delay in clinical detection. To allow for sufficient follow-up evaluation after the initiation of chemotherapy, patients without clinical follow-up data available for at least 12 months after initiation of chemotherapy were excluded. Patients with low- or high-grade appendiceal mucinous neoplasm (LAMN/HAMN) also were excluded because these subtypes are usually managed with surgery rather than chemotherapy.

### Study Outcomes

The primary outcome for the study was disease event-free survival (EFS), defined as the time from cycle 1 day 1 of chemotherapy received for metastatic disease to the date of the earliest occurring disease event of interest. Recurrence of disease after complete cytoreduction (CC0/CC1), progression of disease after incomplete cytoreduction (CC2/CC3), progression in non-surgical cases such that cytoreduction was no longer feasible, or death were considered as disease events of interest for this study. This endpoint was chosen because it is a feasible outcome to measure for both surgical and non-surgical patients.^23^

Surgical intervention categories were pre-specified based on completeness of cytoreduction (CC) scores, with CC0 and CC1 considered “complete” cytoreductions.^24^ Peritoneal carcinomatosis index (PCI) values used in our analysis, when available, were recorded at the time of cytoreduction or use of diagnostic laparoscopy for patients who did not undergo cytoreduction. For patients who received multiple lines of systemic therapy, outcomes after the first chemotherapy received were considered as outcomes, with subsequent lines of treatment perhaps predictably poor and not accurately reflective of EFS for all patients.^25^ The secondary outcome was overall survival (OS) from the date of diagnosis of metastatic disease. Patients who did not record the events of interest for either outcome at the time of review of records were censored at the date of the last available stable imaging (for EFS) and the last clinical contact (for OS).

### Genomic Sequencing

Patients with tumor NGS results available from Clinical Laboratory Improvement Amendments (CLIA)-certified platforms with coverage for *GNAS* were included. Patients with NGS performed at the University of Chicago were sequenced using Oncoplus, our in-house 1212-gene, large-scale, hybrid-capture cancer-sequencing assay. The methodology for testing and variant calling has been described previously.^26^ Most patients (*n* = 37, 77.1 %) had sequencing results available through Oncoplus, whereas 11 (22.9 %) patients had sequencing results available from commercially available NGS platforms or results from NGS performed using another academic medical center’s CLIA-certified in-house assay. Variants of undetermined significance (in *GNAS* as well as other genes) were not considered for our report.

### Statistical Analysis

Statistical analyses were performed using BlueSky Statistics v10.3.2 (BlueSky Statistics LLC, Chicago, IL, USA), and StataNow v18.5 (StataCorp LLC, College Station, TX, USA). Continuous variables are presented as medians with interquartile ranges (IQRs) and were compared using the Wilcoxon test for independent samples. Categorical variables are expressed as proportions and were compared using Fischer's exact test. Kaplan-Meier estimations and Cox proportional hazards regression were used for survival analysis, with proportional hazards assumptions tested by evaluating Schoenfeld residuals.^27–29^ There was no evidence suggestive of violations. Variables with significant associations on univariable Cox regression were included in the multivariable analysis of EFS. Significant variables on multivariable analysis were retained in the final model. Interaction between *GNAS* and *KRAS* variables for EFS and OS models was assessed using a likelihood ratio test comparing the models with and without an interaction term. Co-mutation significance between *GNAS*/*KRAS* was checked using Fischer's exact test. Statistical significance was set at a *p* value lower than 0.05. The genomic landscape was plotted using the Oviz-Bio tool (City University of Hong Kong, Kowloon, Hong Kong SAR).^30^

## Results

**Fig. 1:**
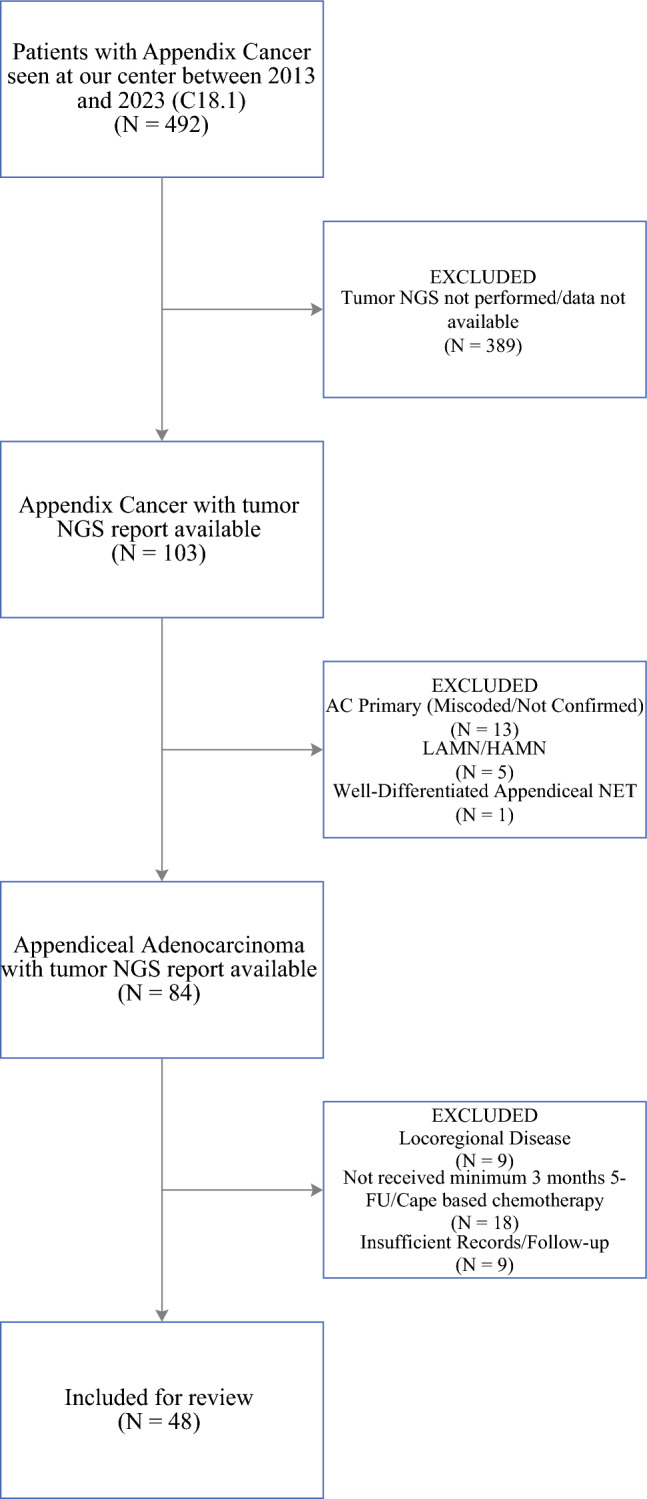
STROBE flowchart depicting the subject selection process. AC, appendiceal cancer; C18.1, ICD10 coding for appendiceal cancer; NGS, next-generation sequencing; LAMN/HAMN, low-grade/high-grade appendiceal mucinous neoplasm; NET, neuroendocrine tumor; 5-FU, 5-fluorouracil; Cape, capecitabine

The study included 48 consecutive patients who had AA seen at the University of Chicago with tumor NGS results available. A brief overview of patient selection is presented in Fig. 1. The median age was 59.3 years (IQR, 51.1–65.2 years). Of the 48 patients, 21 (43.8 %) were female and 40 (83.3 %) had synchronous metastases, whereas 8 (16.7 %) had metachronous metastases. Baseline clinicopathologic characteristics of the study population are described in Table 1.

**Table 1:** Baseline demographics and clinicopathologic characteristics of the study population

Characteristic	No. of patients (% of patients with data available) *n* (%)
Total no. of patients	48
Median age at diagnosis: years (IQR)	59.3 (51.1–65.2)
Sex	
Female	21 (43.8)
Male	27 (56.2)
Race ( % of total)	
White	36 (75)
Black or African American	7 (14.6)
Native Hawaiian/other Pacific Islander	1 (2.1)
More than one	2 (4.2)
Unknown	2 (4.2)
Ethnicity (% of total)	
Hispanic/Latino	1 (2.1)
NonHispanic/Latino	45 (93.8)
Unknown	2 (4.2)
Histology	
Mucinous adenocarcinoma	21 (43.8)
Goblet cell adenocarcinoma	19 (39.6)
Signet ring cell adenocarcinoma	4 (8.3)
Colonic-type (intestinal-type) adenocarcinoma	3 (6.3)
Poorly differentiated adenocarcinoma	1 (2.1)
Lymph node disease	
Present	13 (27.1)
Not present (N0)	20 (41.7)
Could not be assessed (Nx)	15 (31.3)
Lymphovascular invasion present (*n* = 44)	19 (43.2)
Perineural invasion present (*n* = 44)	23 (47.9)
Grade	
1	8 (16.7)
2	15 (31.3)
3	25 (52.1)
Metastases	
Synchronous	40 (83.3)
Metachronous	8 (16.7)
Cytoreductive surgery	34 (70.8)
CC0 reduction achieved (*n* = 34)	15 (44.1)
CC1 reduction achieved (*n* = 34)	11 (32.4)
CC2/CC3 reduction achieved (*n* = 34)	8 (23.5)

IQR, interquartile range; CC, completeness of cytoreduction score

### Therapy Characteristics

Of the 48 patients, 34 (70.8 %) received systemic chemotherapy and underwent concurrent CRS for metastatic/recurrent AA. The most commonly used chemotherapy regimen was FOLFOX (5-fluorouracil, leucovorin, and oxaliplatin) chemotherapy (*n* = 25, 52.1 %), with 13 (27.1 %) patients receiving a triplet chemotherapy regimen (FOLFIRINOX/FOLFOXIRI: 5-fluorouracil, leucovorin, irinotecan, and oxaliplatin), and with the remaining 35 (72.9 %) patients receiving 5-fluorouracil or capecitabine-based doublet regimens. For 24 (50 %) patients, bevacizumab was administered alongside chemotherapy. Chemotherapy was received for a median of 6 months (range, 3–31 months). The therapeutic interventions used in the study cohort are summarized in Table S1.

### Genomic Sequencing

A *GNAS*-activating mutation was detected in 18 (37.5 %) patients, all at the *R201* hotspot (15 at *R201H* and 3 at *R201C*). A comparison of the clinicopathologic characteristics of the group with *GNAS*-activating mutations (*GNAS*^mt^) and *GNAS* wild-type (*GNAS*^wt^) groups is presented in Table 2. Notably, the *GNAS*^mt^ group had significantly smaller proportions of higher-grade disease (*p* = 0.003), lymphovascular invasion (LVI; *p* = 0.005), and perineural invasion (PNI; *p* = 0.03). A significantly higher proportion of *GNAS*^mt^ cases had mucinous histology (*p* < 0.001). A higher median PCI (*n* = 43) was noted in the *GNAS*^mt^ group (*p* = 0.03).

**Table 2:** Clinicopathologic characteristics of the study cohort stratified by status of *GNAS*-activating mutations

	*GNAS*^mt^		*GNAS*^wt^		*p* Value^a^
	*n*	%	*n*	%	
*n*	18		30		
Median age at diagnosis: years (IQR)	58.6 (50.8–66.1)		59.6 (51.8–64.7)		0.87
Sex					0.77
Male	11	61.1	16	53.3	
Female	7	38.9	14	46.7	
Race					0.28
White	15	83.3	21	70	
Non-white/unknown	3	16.7	9	30	
Histology					**< 0.001**
Mucinous Adenocarcinoma	16	88.9	5	16.7	
Non-mucinous Adenocarcinoma	2	11.1	25	83.3	
Grade					**0.003**
1	7	38.9	1	3.3	
2	6	33.3	9	30	
3	5	27.8	20	66.7	
Metastases					0.99
Synchronous	15	83.3	25	83.3	
Metachronous	3	16.7	5	16.7	
Lymph node metastasis (*n* = 33)	2/12	16.7	11/21	52.4	0.07
Lymphovascular invasion present (*n* = 44)	2/15	13.3	17/29	58.6	**0.005**
Perineural invasion present (*n* = 44)	4/15	26.7	19/29	65.5	**0.03**
PCI (IQR; *n* = 43)	26.5 (21.8–31)		19 (12–22)		**0.03**
CRS performed	12	66.7	22	73.3	0.75
CC0/CC1 reduction achieved	8	44.4	18	60.0	0.38
Preoperative chemotherapy received (vs postoperative only; *n* = 34)	9/12	75	17/22	77.3	0.99
*KRAS* mutation	13(*KRAS*/*GNAS* co-mutated)	72.2	9	30	**0.007**
Median months of chemotherapy (IQR)	6 (4–6)		6 (5.25–6)		0.44

mt, mutant; wt, wild type; IQR, interquartile range; PCI, peritoneal carcinomatosis index; CRS, cytoreductive surgery; CC, completeness of cytoreduction score

^a^Significant *p* values are depicted in bold

**Fig. 2:**
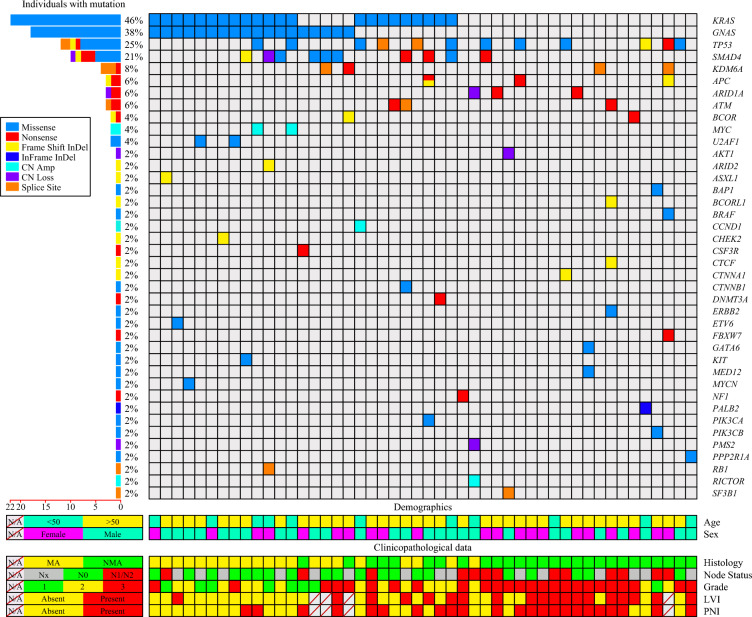
Pictorial depiction of clinicopathologic features with corresponding mutations detected in the cohort. CN Amp, copy number amplification; CN Loss, copy number loss; FrameShift In-Del, frame-shift insertion or deletion; In-Frame InDel, in-frame insertion or deletion;gender; LVI, lymphovascular invasion; PNI, perineural invasion

In our study cohort, *KRAS* (*n* = 22, 45.8 %), *TP53* (*n* = 12, 25 %), *SMAD4* (*n* = 10; 20.8 %), *APC* (*n* = 3; 6.3 %), *ARID1A* (*n* = 3, 6.3 %), *ATM* (*n* = 3, 6.3 %), and *KDM6A* (*n* = 3, 6.3 %) also were frequently mutated (Fig. 2), with *GNAS* and KRAS significantly co-mutated (*p* = 0.007; Table 2).

### Survival Outcomes

**Fig. 3:**
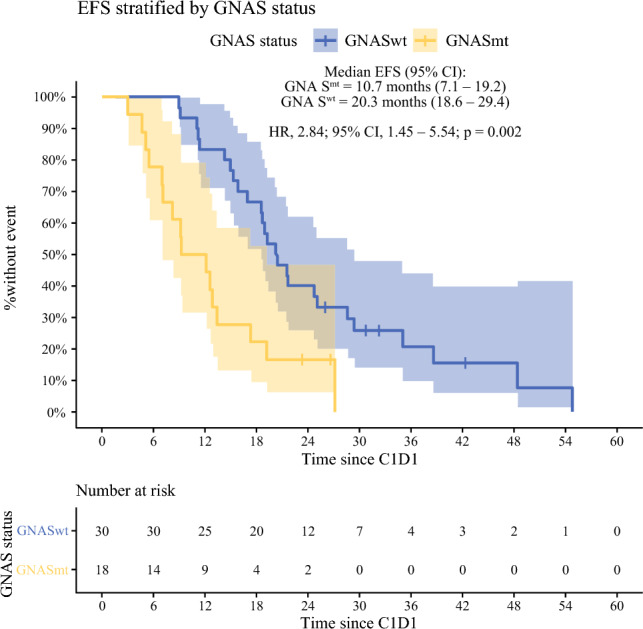
Kaplan-Meier plot for event-free survival calculated from date of cycle 1 day 1 of chemotherapy stratified by *GNAS* mutation status. mt, mutant; wt, wild type; HR, hazard ratio; EFS, disease event-free survival; NB, HR presented is from univariable Cox proportional hazards regression. EFS, time from start of chemotherapy to the earliest disease event: death, clinical/radiographic recurrence, or progression

The presence of a *GNAS*-activating mutation was associated with significantly shorter EFS (10.7 months [95 % CI, 7.1–19.2 months] vs 20.3 months [95 % CI, 18.6–29.4 months]; *p* = 0.002, univariable Cox; Fig. 3). A distribution of disease events based on treatment and GNAS status is depicted in Table S3. The median follow-up period across patients who did not have any recorded disease event of interest (*n* = 6) was 28.7 months (IQR, 26.2–31.9 months). In the multivariable analysis, *GNAS* mutations (adjusted HR [aHR], 3.75; 95 % CI, 1.84–7.63; *p* < 0.001) and metachronous metastases (aHR, 5.14; 95 % CI, 2.08–12.69; *p* < 0.001) were associated with worse EFS. Cytoreductive surgery with both CC0-1 (aHR, 0.12; 95 % CI, 0.05–0.28; *p* < 0.001) and CC2-3 (aHR, 0.28; 95 % CI, 0.10–0.81; *p* = 0.02) was associated with prolonged EFS compared with patients who were not surgical candidates (Table 3).

**Table 3:** Uni- and multivariable analysis for EFS^a^

Variable	Univariable Cox PH results		Multivariable Cox PH results	
	HR (95 % CI)	*p* Value	HR (95 % CI)	*p* Value^b^
Age at diagnosis	1.00 (0.97–1.03)	0.84		
Male vs female	1.44 (0.75–2.78)	0.28		
Non-mucinous vs mucinous histology^c^	0.48 (0.25–0.93)	**0.03**		
Grade				
1	REF			
2	0.73 (0.28–1.92)	0.52		
3	0.57 (0.24–1.38)	0.22		
No. of months of chemotherapy	0.95 (0.85–1.05)	0.30		
Surgical intervention				
No CRS	REF		REF	
CC0-1 cytoreduction	0.16 (0.07–0.35)	**< 0.001**	0.12 (0.05–0.28)	**< 0.001**
CC2-3 cytoreduction	0.24 (0.09–0.67)	**0.006**	0.28 (0.10–0.81)	**0.02**
Bevacizumab vs no bevacizumab use	1.43 (0.77–2.66)	0.26		
Triplet regimen vs doublet regimen	0.91 (0.46–1.79)	0.79		
Metachronous vs synchronous metastasis	2.70 (1.21–6.02)	**0.02**	5.14 (2.08–12.69)	**< 0.001**
Prior chemotherapy for localized disease	1.86 (0.65–5.31)	0.24		
*GNAS*^mt^ vs *GNAS*^wt^	2.84 (1.45–5.54)	**0.002**	3.75 (1.84–7.63)	**< 0.001**

EFS, Event-free survival; PH, proportional hazards; HR, hazard ratio; CI, confidence interval; REF, reference; CRS, cytoreductive surgery; mt, mutant; wt, wild type; CC, completeness of cytoreduction score; mt, mutant; wt, wild type

^a^Variables with a *p* value < 0.05 in univariable analysis were considered for inclusion in multivariable analysis

^b^Significant *p* values are depicted in bold

^c^Non-mucinous histology was subsequently excluded because it was not significant on multivariable Cox regression

In a limited subset with PCI data available (*n* = 43), the association between *GNAS* mutations and EFS remained significant after adjustment for PCI (aHR, 2.37; 95 % CI, 1.09–5.17; *p* = 0.03). *KRAS* mutations (HR, 1.32; 95 % CI, 0.69–2.53; *p* = 0.40), *TP53* mutations (HR, 0.80; 95 % CI, 0.39–1.64; *p* = 0.55), and *SMAD4* mutations (HR, 1.62; 95 % CI, 0.79–3.35; *p* = 0.19) did not show any significant associations with EFS.

Overall survival was analyzed from the date of metastatic disease diagnosis. Patients with *GNAS*^mt^ disease did not have significantly different OS compared with the *GNAS*^wt^ group (40.9 months [95 % CI, 30.2–NR months] vs 35 months [95 % CI, 29.9–47.6 months]) (HR, 0.68; 95 % CI, 0.31–1.47; *p* = 0.33). However, our OS comparison might have been underpowered to detect a significant difference between the groups given the observed effect (HR, 0.68). A sensitivity analysis showed that with the available sample size (*n* = 48; 33 events), the study achieved 80 % power (at α ≤ 0.05) only for detecting a minimum detectable effect of HR ≤0.38. Notably, having a *KRAS* mutation was associated with significantly prolonged OS (47 months [95 % CI, 31.1–NR months] vs 31.6 months [95 % CI, 26–43.2 months) on univariable analysis (HR, 0.46; 95 % CI, 0.22–0.98; *p* = 0.04).

Likelihood ratio tests showed no significant multiplicative interaction between the *GNAS* and *KRAS* variables for EFS (*p* = 0.06) or OS (*p* = 0.10). Due to the influence of *KRAS* mutations on survival outcomes, we subsequently performed multivariable Cox regression with both genes included for OS and EFS. The results are shown in Table S2.

## Discussion

Our findings suggest that the presence of *GNAS*-activating mutations may have a predictive role in risk stratification before systemic chemotherapy in patients with metastatic/recurrent AA, and add to the growing body of evidence suggesting that the genomic characteristics of tumors could influence clinical outcomes in patients with appendiceal cancers.^8,19^

The presence of *GNAS* mutations was associated with favorable disease characteristics such as lower grade as well as absence of LVI and PNI involvment in our cohort. This is in line with previous data showing enrichment of *GNAS* mutations in lower-grade appendiceal tumors.^19^ Our study, however, was unique in its inclusion of patients with histologically confirmed AA only, unlike previous reports that have included patients with PMP of unconfirmed origin.^19^ Prior analyses of secondary data have shown a higher frequency of *GNAS* mutations in older patients with AC,^20^ but we did not find any significant difference in the median age at diagnosis in our cohort when it was stratified by *GNAS* mutation status. Our cohort’s being composed of exclusively adenocarcinoma variants could explain this discordance with previous analyses.

Despite favorable histopathologic characteristics, we found significantly reduced EFS, which we considered as a proxy outcome for chemotherapy responsiveness in patients with *GNAS*-mutated tumors when treated with systemic chemotherapy, an association that remained significant after adjustment for CRS.

Associations between the presence of *GNAS* mutations and reduced responsiveness to systemic chemotherapy have been noted in prior studies.^8,21^ However, previous analyses have either included patients with PMP^21^ or reported responsiveness in the form of radiographic disease response.^8^ We believe our study is the first of its kind to assess the predictive role of *GNAS* mutations in AA by assessing survival outcomes using EFS as a marker of treatment benefit.^31^ Because clinical decision-making regarding escalations, modifications, or terminations of systemic therapy are often guided by the disease events included in our definition of EFS, our data are meaningful additions to the current literature in the field.

Patients with *GNAS* mutations showed a significantly higher median PCI. This is consistent with previous reports of the biology and clinical impact of *GNAS-*activating mutations.^8,18,32,33^ Notably, we found that the association between *GNAS* mutations and EFS remained significant after adjustment for PCI, albeit limited to the subset of cases with PCI recorded in our cohort. However, our PCI-related findings may have partly been a result of biases due to preoperative decision-making. Stricter PCI cutoff values are often applied to patients with less favorable tumor characteristics, which may have influenced the findings presented. Furthemore, differences in the timing/method of PCI assessment across our cohort (at time of CRS vs diagnostic laparoscopy) should be taken into account while interpreting our PCI findings. Due to the interplay of *GNAS* activation and cellular-signaling cascades (e.g., mitogen-activated protein kinase [MAPK], Wnt-signaling pathway [Wnt]), mitogen-activated protein kinase kinase [MEK] inhibitors may hold clinical relevance for neoplasms harboring *GNAS*-activating mutations.^34^ Clinical benefit with the use of trametinib, a MEK inhibitor, and palbociclib, a cyclin-dependent kinase 4/6 inhibitor, has been reported in GNAS-murated neoplasms.^35–37^ Hence, targeted therapies may offer superior efficacy over traditional systemic therapy, especially in lower-grade appendiceal adenocarcinomas, in which disease biology is characterized by frequent *GNAS*-activating mutations.

Our study had some limitations. The limited sample size and the single-center retrospective nature of our data precluded reliable subgroup analyses and may diminish the external validity of our findings. Furthermore, negative results, especially in our OS findings, could be a result of our limited sample size rather than a true lack of difference between the groups. Although we used EFS as a surrogate for chemotherapy responsiveness and treatment benefit, several patients underwent CRS, which may influence the utility of EFS in this regard. Non-quantifiable metrics related to patient selection for tumor genomic-profiling could have resulted in ascertainment bias.

Despite these limitations, we believe our report describes a clinically relevant difference in therapeutic efficacy associated with *GNAS* mutations in patients with AAs treated with systemic chemotherapy. Prospective and larger scale validations of these findings are warranted.

## Conclusion

Activating mutations in *GNAS* are associated with worse outcomes with standard systemic therapies used in the management of appendiceal adenocarcinoma. Further investigations to validate the utility of *GNAS* mutations as predictive biomarkers to optimize systemic treatment strategies for these patients are warranted. Novel agents with patient selection based on genomic biomarkers represent an unmet need toward personalizing therapy in appendiceal cancers.

## Supplementary Information

Below is the link to the electronic supplementary material.

Supplementary file1 (DOCX 15 KB)
