# Supplementary material for: Association of Activating GNAS Mutations and Outcomes with Chemotherapy in Metastatic Appendiceal Adenocarcinoma
Source: Ann Surg Oncol. 2026 Feb 3;33(4):3453–61. doi: 10.1245/s10434-025-18805-5 (PMC12982240; doi:10.1245/s10434-025-18805-5)
Supplement: Supplementary file 1 — Supplementary file1 (DOCX 15 KB) [file 10434_2025_18805_MOESM1_ESM.docx]

| **TABLE S1** Therapies received *n* (%) | |
| --- | --- |
| Systemic therapy (chemotherapy) only (% of total) | **14 (29.2)** |
| CRS performed (% of total) | **34 (70.8)** |
| HIPEC administered alongside CRS (*n* = 34) | 29 (85.3) |
| Timing of chemotherapy in *GNAS*^mt^ group among patients who underwent CRS (*n* = 12) | |
| Pre-operative only | 3 (25) |
| Peri-operative (pre-operative and post-operative both) | 6 (50) |
| Post-operative only | 3 (25) |
| Timing of chemotherapy in *GNAS*^wt^ group among patients who underwent CRS ( *n* = 22) | |
| Preoperative only | 9 (40.9) |
| Perioperative (pre- and postoperative both) | 8 (36.4) |
| Postoperative only | 5 (22.7) |
| Chemotherapy regimens used | |
| 5-Fluorouracil, leucovorin, and oxaliplatin | 25 (52.1) |
| 5-Fluorouracil, leucovorin, irinotecan, and oxaliplatin | 13 (27.1) |
| 5-Fluorouracil, leucovorin, and irinotecan | 7 (14.6) |
| Capecitabine and oxaliplatin | 2 (4.2) |
| Capecitabine and irinotecan | 1 (2.1) |
| Bevacizumab use | |
| Bevacizumab used (alongside chemotherapy) | 24 (50) |
| CRS, cytoreductive surgery; HIPEC, heated intraperitoneal chemotherapy; mt, mutant; wt, wild type | |

| **TABLE S2** Multivariable analyses for EFS and OS including *GNAS* and *KRAS* variables | | |
| --- | --- | --- |
| Variable | Multivariable Cox PH results | |
|  | HR (95 % CI) | *p* Value^a^ |
| EFS | | |
| *GNAS*^mt^ vs *GNAS*^wt^ | 3.52 (1.51–8.20) | **0.004** |
| *KRAS*^mt^ vs *KRAS*^wt^ | 0.70 (0.31–1.59) | 0.4 |
| OS | | |
| *GNAS*^mt^ vs *GNAS*^wt^ | 0.99 (0.41–2.36) | 0.98 |
| *KRAS*^mt^ vs *KRAS*^wt^ | 0.47 (0.20–1.09) | 0.08 |
| EFS, event-free survival; OS, overall survival; PH, proportional hazards; HR, hazard ratio; CI, confidence interval; mt, mutant; wt, wild type NB<AQ8>  ^a^Significant *p* values are depicted in bold. | | |

| **TABLE S3** Distribution of events for EFS by groups stratified by mutation status | |
| --- | --- |
| Disease event distribution in *GNAS*^mt^ group among patients who received chemotherapy alone (*n* = 6)^a^ *n* (%) | |
| Disease progression precluding cytoreduction | 6 (100 %) |
| Death | 0 |
| Censor (no event) | 0 |
| Disease event distribution in *GNAS*^mt^ group among patients who underwent CRS (*n* = 12)^a^ | |
| Disease recurrence after CC0/CC1 cytoreduction (*n* = 8)^a^ | 6 (50 %) |
| Disease progression after CC2/CC3 cytoreduction (*n* = 4)^a^ | 4 (33.3 %) |
| Death | 0 |
| Censor (no event) | 2 (16.7 %) |
| Disease event distribution in *GNAS*^wt^ group among patients who received chemotherapy alone (*n* = 8)^a^ | |
| Disease progression precluding cytoreduction | 6 (75 %) |
| Death | 2 (25 %) |
| Censor (no event) | 0 |
| Disease event distribution in *GNAS*^wt^ group among patients who underwent CRS (*n* = 22)^a^ | |
| Disease recurrence after CC0/CC1 cytoreduction (*n* = 18)^a^ | 11 (50 %) |
| Disease progression after CC2/CC3 cytoreduction (*n* = 4)^a^ | 2 (9.1 %) |
| Death | 5 (22.7 %) |
| Censor (no event) | 4 (18.2 %) |
| EFS, event-free survival; CRS, cytoreductive surgery; CC, completeness of cytoreduction score  ^a^Number.in parentheses represents total number of patients in each subgroup. | |
